# State Examinations for License

**Published:** 1894-11

**Authors:** 


### STATE EXAMINATIONS FOR LICENSE.

### PENNSYLVANIA.

An autumn examination will be held by the Pennsylvania Board. Its influence is already clearly felt in the solicitude shown by some of the medical schools concerning the fitness of students to pursue the study of medicine. It was rare in former years to hear the dean of a medical school advise a pupil not to enter or continue in the course unless, indeed, the student had paid all the fees that the college could exact. We hear now, however, occasionally of students being advised that they are not sufficiently prepared to enter upon the medical course. It is to be hoped that the Pennsylvania Board will maintain the position that was assumed in the first examination. The questions were highly satisfactory. They were such as any well-educated graduate could answer, and if perfect impartiality, fairness and strictness are maintained, the effect upon the colleges and upon the profession will be most beneficial.—*Medical News*, October 13, 1894.

### NEW YORK.

We publish the following statistics of the September examinations for state medical license by the board representing the Medical Society of the State of New York :

| | | |
|---|---|---|
| Total number of candidates | 76 | |
| Successful | 51 | |
| Unsuccessful | 25 | |
| Percentage of rejections | 32.9 | |
| Rejected in anatomy | 7 | |
| " " physiology and hygiene | 3 | |
| " " chemistry | 6 | |
| " " surgery | 9 | |
| " " obstetrics | 16 | |
| " " pathology and diagnosis | 10 | |
| " " practice, materia medica and therapeutics | 6 | |
| | 57 | |
| Average rejections in each topic | 8 | |
| Rejected in one topic | 12 | 12 |
| " " two topics | 3 | 6 |
| " " three " | 5 | 15 |
| " " four " | 3 | 12 |
| " " five " | 1 | 5 |
| " " seven " | 1 | 7 |
| | | 57 |

In connection with the above statistics we would invite special attention to the address of Prof. F. W. Hinkel, M. D., published in this issue of the JOURNAL, which deserves to be read by every medical student in the State of New York. If the several colleges in this state, following the example of Dr. Hinkel, would instruct their pupils on the subject of state control of medical license, it would serve to increase the general intelligence on the subject and to better prepare students for the final examination by the state.

